# Perceived Mental Workload Classification Using Intermediate Fusion Multimodal Deep Learning

**DOI:** 10.3389/fnhum.2020.609096

**Published:** 2021-01-11

**Authors:** Tenzing C. Dolmans, Mannes Poel, Jan-Willem J. R. van ’t Klooster, Bernard P. Veldkamp

**Affiliations:** ^1^Data Management and Biometrics, University of Twente, Enschede, Netherlands; ^2^Behavioural, Management and Social Sciences Lab, University of Twente, Enschede, Netherlands; ^3^Research Methodology, Measurement, and Data Analysis, University of Twente, Enschede, Netherlands

**Keywords:** brain-computer interface (BCI), deep learning, multimodal deep learning architecture, device synchronisation, fNIRS (functional near infrared spectroscopy), GSR (galvanic skin response), PPG (photoplethysmography), eye tracking (ET)

## Abstract

A lot of research has been done on the detection of mental workload (MWL) using various bio-signals. Recently, deep learning has allowed for novel methods and results. A plethora of measurement modalities have proven to be valuable in this task, yet studies currently often only use a single modality to classify MWL. The goal of this research was to classify perceived mental workload (PMWL) using a deep neural network (DNN) that flexibly makes use of multiple modalities, in order to allow for feature sharing between modalities. To achieve this goal, an experiment was conducted in which MWL was simulated with the help of verbal logic puzzles. The puzzles came in five levels of difficulty and were presented in a random order. Participants had 1 h to solve as many puzzles as they could. Between puzzles, they gave a difficulty rating between 1 and 7, seven being the highest difficulty. Galvanic skin response, photoplethysmograms, functional near-infrared spectrograms and eye movements were collected simultaneously using LabStreamingLayer (LSL). Marker information from the puzzles was also streamed on LSL. We designed and evaluated a novel intermediate fusion multimodal DNN for the classification of PMWL using the aforementioned four modalities. Two main criteria that guided the design and implementation of our DNN are modularity and generalisability. We were able to classify PMWL within-level accurate (0.985 levels) on a seven-level workload scale using the aforementioned modalities. The model architecture allows for easy addition and removal of modalities without major structural implications because of the modular nature of the design. Furthermore, we showed that our neural network performed better when using multiple modalities, as opposed to a single modality. The dataset and code used in this paper are openly available.

## Introduction

Mental workload (MWL) has gained a lot of attention in a variety of fields, such as neuroscience (Toppi et al., 2016; Lim et al., 2018), human factors and ergonomics (Schmalfuß et al., 2018) and human factors in computing systems (Duchowski et al., 2018). In the context of this work, MWL depends on two variables: available cognitive resources and required cognitive resources. Determining the available cognitive resources requires information about prior knowledge, ability and task experience and is thus highly personal. The required cognitive resources depend on task difficulty. In a state of “flow,” as described by Csikszentmihalyi (1975), one experiences full emersion with the task at hand. In such a state, the ratio between the available and required cognitive resources, or *α*, is between 0.8 and 1.2 (Csikszentmihalyi, 1997). The ability to approximate *α* is interesting, since it would yield insight into MWL and allow for adaptations of tasks. Typically, participants are actively involved in (self)assessing their MWL. The NASA Task Load Index (NASA-TLX) questionnaire is often used to retrieve information about the magnitude and sources of six workload-related factors (Hart and Staveland, 1988). Explicitly acquired information about MWL through retrospection is subjective and results in a measure of perceived mental workload (PMWL). The mere act of performing a measurement on a phenomenon can interfere with the phenomenon (Mahtani et al., 2018). Hence, requiring subjects to extensively reflect and report on their PMWL during experimentation will impact objectivity, not to mention interrupt their state of flow. Physiological measurements can provide an alternative to repeated self-assessment; an advantage of such bio-signals is that they can be measured implicitly. They can objectively be acquired in real-time without explicitly asking participants to provide this data.

The classification of PMWL has been attempted in a unimodal setting using various physiological signals, such as functional near-infrared spectroscopy (fNIRS; Shin et al., 2018), galvanic skin response (GSR; Nourbakhsh et al., 2017) and heart rate (HR), through photoplethysmography (PPG; Schmalfuß et al., 2018). All the aforementioned modalities have individually proven to be useful for the classification of PMWL. This research sought to use an advantageous approach to the classification of PMWL by leveraging both information inherent in individual modalities, as well as cross-modality information. Fusion-based approaches have been surveyed in Baltrušaitis et al. (2018), covering a.o. multi-layer multimodal fusion (Vielzeuf et al., 2018), attention-based methods (Hori et al., 2017) and correlation neural networks (Chandar et al., 2016). Our primary objective in this study is, however, not to give a literature overview, but to actually classify PMWL. The secondary objective is to determine what physiological signals provide valuable information about PMWL. First, we formulated design principles that are relevant and effective within the context of multimodal signal classification that makes use of deep learning. To achieve the primary objective, these design principles were used in the formulation of an intermediate fusion multimodal network (IFMMoN).

## Materials and Methods

Our goal was to classify PMWL using a deep neural network (DNN) that flexibly makes use of multiple modalities. During the design of such a multimodal brain–computer interface, the principles used to design the end-to-end data path, or pipeline, guide the outcome. Two key aspects of the pipeline were modularity and generalisability (MG). To be modular, new devices should be easy to add to the setup, and their data (collection and processing) should fit within the pipeline with minimal structural implications. Two important libraries that aided modularity throughout the research were used: LabStreamingLayer (LSL) and TensorFlow. LSL provided modularity by allowing device-specific data streams to be easily added (Kothe, 2014). The TensorFlow API allowed for modularity in deep learning model creation (Abadi et al., 2016). Generalisability implies that the additional data that become available from added modalities contribute to classification accuracy. To further improve generalisability and thus applicability, the pipeline should also function well in the classification of other topics besides PMWL. The MG criteria require our methods to be circumstance and device independent where possible. Serendipitously, they served as a way of reducing human error by automating much of the data gathering and analysis pipelines. All methods and designs applied in this project were formulated and executed with the MG criteria in mind.

In the first part of this section, we look to previous works in the field to determine approaches for each of our modalities, as well as fusion options of the DNN. From there, we discuss stimulus presentation, participants and data collection and synchronisation. Lastly, model optimisation with the help of the TensorFlow and Optuna toolboxes is discussed (Abadi et al., 2016; Akiba et al., 2019).

### Related Work

We combined a total of four modalities to classify PMWL using this novel approach. To record brain activity, we opted for fNIRS to measure change in (de)oxygenation in the brain (Villringer et al., 2013). Our method is based on Shin et al. (2018). Other bio-signals that we measure are GSR, based on Nourbakhsh et al. (2017), and HR using PPG, on the basis of Schmalfuß et al. (2018). Lastly, eye tracking (ET) was also done, as inspired by Duchowski et al. (2018). In the following subsections, we discuss these modalities in more detail. Then, we discuss what deep learning methods have previously been used to process their data. Fusion options for the combination of data from various devices are finally discussed.

#### Functional Near-Infrared Spectroscopy

Through fNIRS, relative changes in (de)oxyhaemoglobin concentrations in the brain can be measured. During activation of brain function, energy use and thus the distribution of haemoglobin change (Villringer et al., 1993). This change can be measured using near-infrared light and then be correlated with activation in specific regions of tissue. It seems that there exists no clear consensus about the “best” deep learning-based analysis method for fNIRS data in MWL detection. Literature can be divided into two main categories: Multilayer Perceptrons (MLPs), consisting of several densely connected layers, and Convolutional Neural Networks (CNNs). Though less common, Recurrent Neural Networks (RNNs) were also used for processing fNIRS (Zhao et al., 2019). Some authors who opted for generic MLPs (e.g., Naseer et al., 2016; McDonald and Solovey, 2017) show great accuracy on binary problems, as well as on more complicated problems. The papers report 63% accuracy on user identification (*n* = 30; McDonald and Solovey, 2017) and over 91% in binary classification of mental arithmetic vs. rest (Naseer et al., 2016). While the former accuracy is seemingly low, the objective of classification in the work of McDonald and Solovey (2017) is much more natural since the authors’ objective was to do user identification on the basis of recorded data. They reported that among 30 subjects, they could determine what data the participant belonged to with a 63% accuracy, whereas chance level is 3.3%.

Tanveer et al. (2019) used two models in their work: one for Beer–Lambert modified optode densities and another for heatmaps of channels over time. Their first network was a DNN with six fully connected dense layers, and their second was a CNN with two convolutional layers and two dense layers. Binary cross-entropy loss was used as loss measure. They report an accuracy of 99.3% on binary classification, achieving the best result with the CNN. Dargazany et al. (2019) showed that an accuracy of over 80% can be reached in 5-class motor imagery problem using a MLP. The benefit of their approach is that they did not perform any pre- or post-processing to the data. This makes their solution very scalable in terms of required human attention, since the majority of the time invested by future users of the system is spent on the collection of data, rather than the (pre)processing of it. However, to facilitate this, their network used two fully connected layers with 10,000 neurons each, leading to quite serious computational complexity.

#### PPG and GSR

PPG is an optical method for measuring blood volume changes in microvascular tissues and is directly related to cardiac activity (Selvaraj et al., 2008). As such, it can be used to measure HR and compute measures, such as HR variability and inter-beat intervals. Biswas et al. (2019) demonstrated that an accuracy of over 95% can be reached on a HR classification task where the goal was to perform biometric identification of users. They propose using two convolutional layers in conjunction with two long short-term memory (LSTM) layers, followed by a dense output layer. GSR is an electrodermal response that is associated with the innervation of the sympathetic nervous system that is often used to measure affective and cognitive arousal (Venables and Christie, 1980). Sun et al. (2019) showed that a LSTM–CNN hybrid network can reach up to 74% accuracy in a six-class emotion recognition problem using GSR. The use of LSTM is attractive in GSR for several reasons: the time domain and temporal nature of the data enables the extraction of metrics, such as peak frequency and amplitude (Nourbakhsh et al., 2017).

Both PPG and GSR can be processed using methods that are focussed on feature extraction. The benefit of working with such features is that they are easy and cheap to compute. However, such feature extraction removes hidden features that may be found by a DNN and negates the possibility of serendipitous findings when combined with other modalities. Besides the above described methods, both modalities can also conveniently be processed with fully connected layers due to their unidimensional shape.

#### Eye Tracking

ET is used to gain information about where a person is looking at any given time, which can help understand visual- and display-based information processing (Poole and Ball, 2006). The training and evaluation of ET data are highly task dependent; therefore, this section does not contain any statements about achieved accuracies and will only discuss the types of networks that are used in the literature. Louedec et al. (2019) use a CNN to predict saliency maps in chess games. Their model is based on VGG16, which was first introduced by Simonyan and Zisserman (2014). Furthermore, their model comprises several deconvolutional layers and fusion layers. Krafka et al. (2016) also use convolutional layers and combine them with fully connected layers. In their work, they classified gaze based on an input face-grid that contains the location of the face, the right and left eyes as well as the full face. Generally, the consensus is to use convolutional layers for the classification of ET data, regardless of objective. Intuitively, this makes sense since we are interested in spatial features in the data.

#### Fusion Options

There exist many strategies to tackling the multimodal problem in deep learning. Given that most neural networks are highly task dependent, the design of a multimodal DNN follows this same trend. Ramachandram and Taylor formulated several key considerations to be made for deep learning with multiple modalities in their overview of deep multimodal learning (Ramachandram and Taylor, 2017). The first key consideration is when to fuse the modalities. In general terms, there exist three options for the time of fusion. The first is early fusion, or data level fusion. This can, for example, be achieved by concatenating features or raw data and feeding said data into a neural network. The second is intermediate fusion. This involves mapping input to a lower dimension using various types of layers and fusing somewhere along the way between the input and output layers. The third option is late fusion, through e.g., majority vote of several smaller networks. The choice of where fusion takes place is flexible and immensely impactful on model performance, as demonstrated by Karpathy et al. (2014). The second key consideration is which modalities to fuse, since not all data contribute to solving a problem equally. The third and final consideration to make is what to do with missing modalities or data. The absence of data can be prohibitively problematic, especially in real-time applications.

In light of MG, early fusion is an unattractive option: it requires the input data to be “stitched” together, which leads to multiple problems in our application. First, we are working with vastly different sampling rates ranging from 10–256 Hz. Furthermore, the dimensionality differs between devices, requiring us to devise a strategy that would guarantee equal share of data in each sample without losing any features that are present in either temporal or spatial dimensions. Lastly, instead of working with an MG network, all concatenated data would be fed into the same network, regardless of what devices are featured in the data. This would entail tuning early layers and shapes of the network when the parameters of the data change. Late fusion through majority vote aligns with the modularity requirement, but not the generalisability requirement. Adding or removing modality networks, or MNets, does not require the adaption of other MNets. However, separated networks are unable to learn from multiple modalities simultaneously, since there is no information exchange between them. Intermediate fusion allows for the creation of several modular MNets that develop “expertise” in their respective domain. This expertise can then be shared with an overarching network. Furthermore, adding or removing modalities is as simple as “clipping on” MNets, or switching them off in the head class, respectively. Hence, intermediate fusion satisfied the MG criteria best.

### Stimulus Presentation

**Figure 1:**
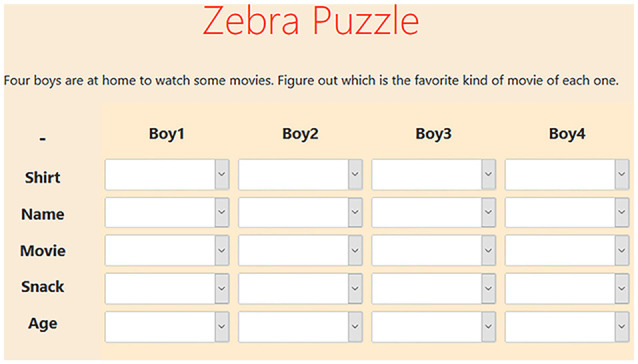
Example zebra puzzle. Below the puzzle, several hints are given that allow participants to connect all attributes (vertical) to each boy (horizontal). An example hint is: “Joshua is in one of the ends.” Clicking the arrow of a cell drops down all options for that cell.

To simulate MWL, participants were asked to solve several zebra puzzles. Zebra puzzles are verbal logic puzzles that are solved by connecting attributes to objects on the basis of hints. The difficulty of the puzzle was modulated by the number of hints that were given and the average number of hints required before an attribute could be chosen. Hints could be ticked off when used. Figure 1 provides an example of a zebra puzzle. In total, there were five different puzzles, each with their own difficulty ranging from “very low” to “very high” difficulty. All puzzles were retrieved from Brainzilla (2020), and initial difficulty indications were also based on the content of Brainzilla. Between every puzzle, participants were asked to take a moment to relax. Furthermore, they indicated how difficult they perceived the puzzle to be on a scale of one to seven, seven being the highest difficulty. These ratings were later used as labels during training. The order in which the puzzles were presented was completely randomised. An LSL stream was active during the entirety of the stimulus presentation. This stream sent a marker at every action. Actions were (un)selecting hints and (un)selecting answers. Markers contained the participant ID, action timestamp, type of action, the id of the action and status of the action (correct, incorrect or checked). The timestamps of this stream are later used to segment the data.

### Participants

In total, data were collected from 23 participants (11 males, 12 females, mean age = 24.7, SD = 9.8, min = 20, max = 57). Of these participants, one was excluded from the dataset because of poor data quality. Participants were recruited using the Sona system, a cloud-based participant management software (SonaSystems, 2020) that is used at the University of Twente. Recruitment was also done in social circles. Prior to experimentation, the study was approved by the ethics committee of the BMS faculty of the University of Twente. All participants granted written informed consent for the collection and open sourcing of data.

### Data Collection and Synchronisation

All data were streamed and recorded on a Dell Precision 3530 Laptop with an Intel i7–8750H CPU, 16 GB RAM and an NVIDIA Quadro P600 GPU. Three different devices measured four modalities. The Shimmer3 GSR+ was used to measure GSR and PPG (Shimmer GSR3+; Shimmer, Dublin, Ireland), the Tobii Pro X3–120 was used for ET (Tobii X3–120; Tobii Group, Stockholm, Sweden) and the Brite^24^ was used to collect fNIRS (Brite^24^; Artinis Medical Systems, Elst, The Netherlands). Since participants were aware of sensors that were attached to their bodies, measurements were not unobtrusive. Each device was set up such that data streams were sent to LSL in real-time. The LabRecorder app was used to record data from all streams into a single XDF file per participant (Kothe, 2014). Data were then imported into Python using PyXDF (Boulay, 2020), which automatically performs checks on the indicated vs. received sampling rates and de-jitters the data where necessary. Data synchrony was also checked manually to ensure that all streams were aligned throughout the recording. Several checks for synchrony were also implemented during data selection and processing, which are documented in the “Data Selection” section.

**Figure 2:**
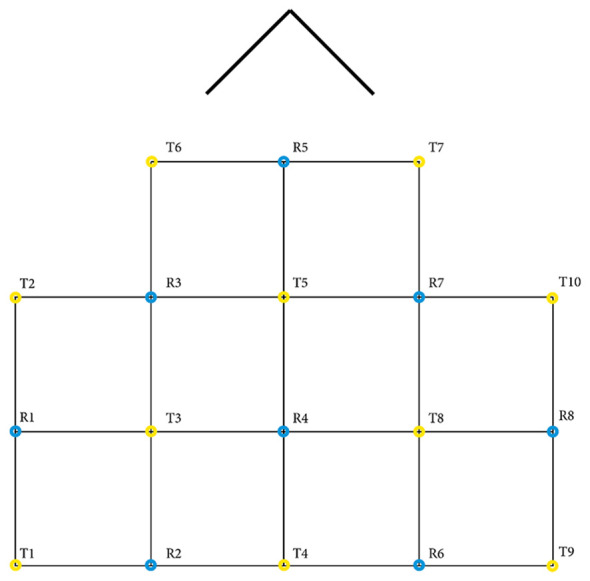
Optode template for fNIRS collection. Between each transmitter (T) and receiver (R), one channel exists. In total, there are 10 transmitters, eight receivers and 27 channels. The arrow represents the nose of the participant. This Brite^24^ 27 channels optode arrangement is available in Oxysoft 3.2.51 (OxySoft; Artinis Medical Systems, Elst, the Netherlands).

**Figure 3:**
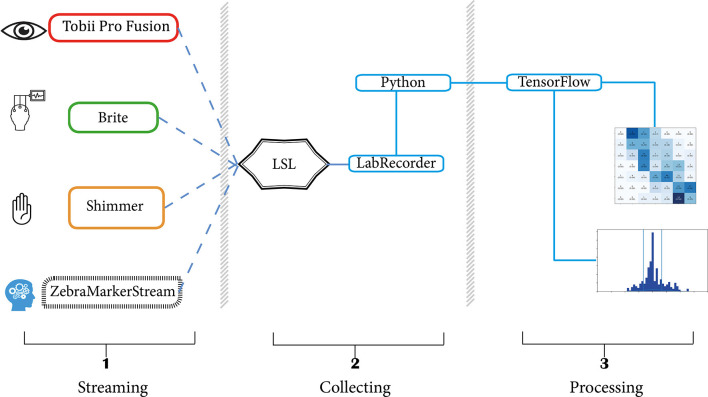
Experimental setup. The left section contains an overview of all active data streams, being: Tobii Eye Tracking data, Brite fNIRS data, Shimmer GSR and PPG data, and the Zebra Puzzle marker stream. Dotted lines indicate a streamed connection to LSL. The LabRecorder in the middle section records the data in XDF format. The right section shows example outputs of processed data.

Raw GSR and PPG were directly streamed to LSL from the Shimmer3 GSR+ using an application that was written by the HBA Lab of Thales (Groot de, 2020). The sampling rate of this stream was 256 Hz. The data of the Tobii Pro X3–120 were streamed using a custom python application that was made with the Tobii Pro SDK and PyLSL (Kothe, 2014; TobiiProAB, 2019). ET data were streamed at 120 Hz and contained x and y coordinates for both eyes. For the collection of fNIRS, Oxysoft 3.2.51.4 × 64 was used (OxySoft; Artinis Medical Systems, Elst, The Netherlands) with the Brite^24^ in the available 27 channels optode arrangement. Two wavelengths (756 and 853 nm) were sampled at 10 Hz, and the Beer–Lambert modified optode densities of O_2_Hb and HHb were mapped to LSL directly from Oxysoft. See Figure 2 for a detailed view of the optode template. For a complete overview of the data pipeline, please refer to Figure 3.

### Model Optimisation

#### Data Selection

Data were selected on the basis of markers that were present in the Zebra Puzzle’s data stream. These markers were sent through LSL at a variable rate that depended on the participant’s actions. The nature of the stimulus presentation contributed to several key things to pay attention during data selection. For one, markers could be close together when participants selected multiple answers in quick succession. Hence, selections made around these markers contained some overlap in data. Due to software issues and practical shortcomings, some parts of data were missing. To overcome these problems, several Boolean masks determined which markers were fit for usage. First, the nearest index to the time of a marker was identified in the data of each device. When said indices were identical for multiple markers, the samples were removed from the dataset. Such exactly matching indices were likely the result of drifting device timestamps and/or missing data and were hence excluded. Segmented selections were inspected for noise by means of computing simple statistics of samples, such as mean, variance, max, min, etc., to gain an overview of data quality. However, noisy samples exposed the network to ‘realistic’ data and were not removed thusly.

Once a full selection of the markers was made, a segment of 8 s of data before the marker was selected; 8 s, because the haemodynamic response function shows a peak after 5–8 s of neuronal activity onset (Zhang et al., 2005); before, because the participant’s contemplation takes place prior to knowing and selecting the correct answer. A CSV file that contained the final selection of the markers was created for each participant. Each sample in our dataset consists of four synchronised measurements: fNIRS, GSR, PPG and ET. The difficulty rating for the sample’s respective puzzle served as the label. These samples were added to a TFRecord file, which allowed many useful methods, such as shuffling, batching and splitting, to be applied to all the selected data simultaneously (TensorFlow, 2020).

The dataset that was generated and analysed for this study can be found in the 4TU.ResearchData repository under the following doi: 10.4121/12932801 (Dolmans et al., 2020).

#### Models

**Figure 4:**
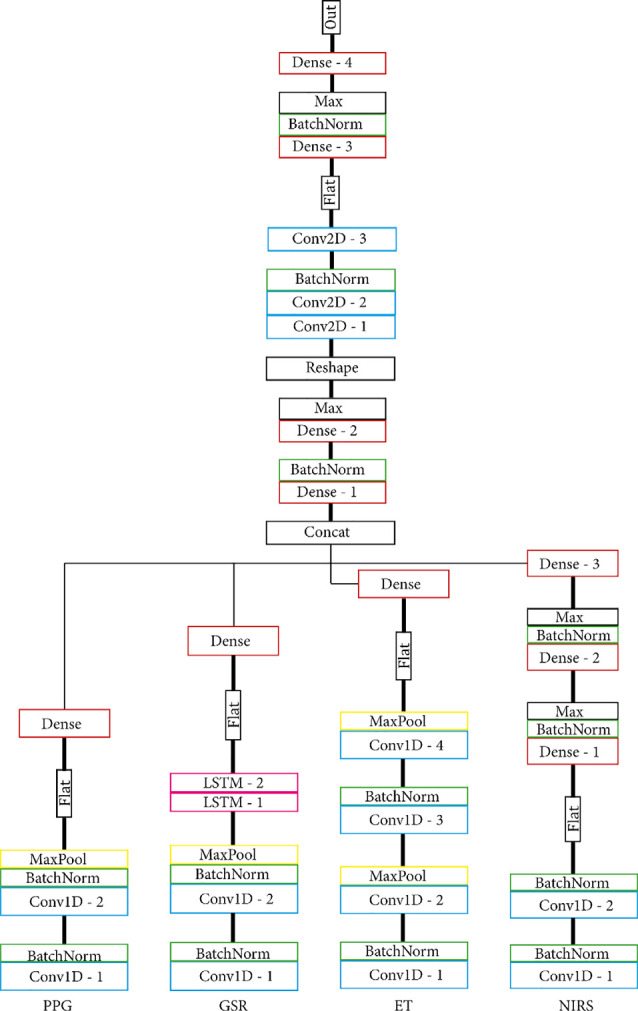
(S_) LIT model. Each modality is indicated at the bottom. All MNets feed into one Head network and are based on what is commonly used in the literature. Before fusion in the Head network, MNets are flattened and represented in a lower-dimensional space with the help of a single densely connected layer.

**Figure 5:**
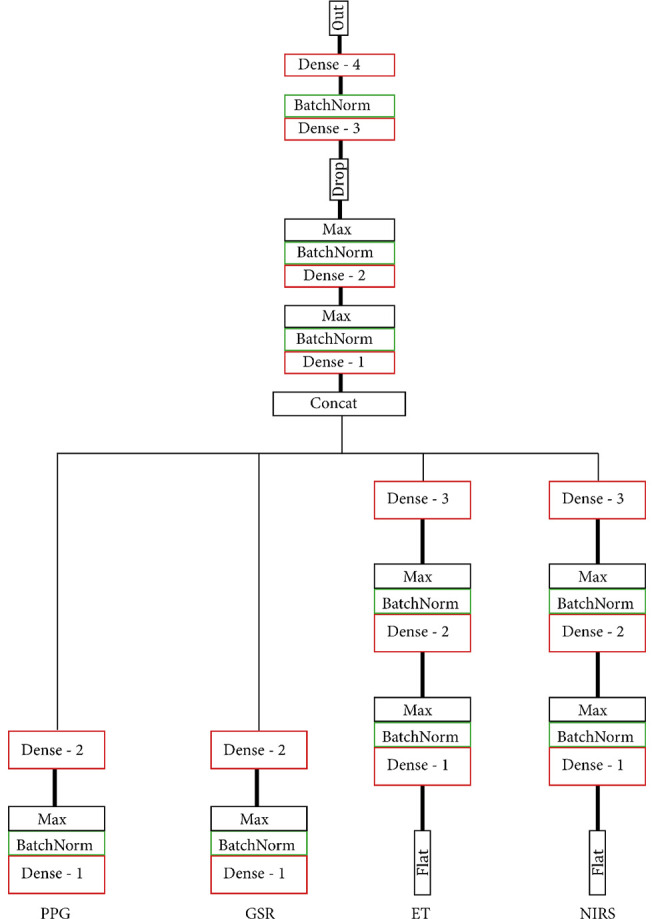
(S_) MLP model. Each modality is indicated at the bottom. All MNets feed into one Head network. Both the MNets and the Head consist solely of densely connected layers.

To maximise usability and allow for feature sharing of our multimodal data while also adhering to the MG criteria, we opted for intermediate fusion. This resulted in a model architecture that adheres to the general structure of one base network, or MNet, for each modality and one Head network that integrates all MNets. Two concrete routes were chosen for the implementation of both the MNets and the Head networks: one model based on literature and one model that contains only densely connected layers. The model based on previous work as discussed in the “Related Work” to “Participants” sections had four custom MNets, one for each modality and one custom Head network. The PPG MNet consisted of two convolutional layers; the GSR MNet consisted of two convolutional and two LSTM layers; the ET MNet consisted of four convolutional layers; finally, the fNIRS MNet consisted of two convolutional and two dense layers. All MNets were represented in a lower-dimensional space with the help of a single densely connected layer before fusion in the Head. Figures 4, 5 contain the structure and layers of the model based on literature and the densely connected model, respectively. Table 1 details the models’ layers and the number of units/filters for each layer. Batch normalisation and max pooling were utilised as a means of stabilisation. For both models, a smaller alternative model was created that contained exactly half of the units and filters in each layer in order to gain an initial idea of the effect of reduced network size. This brought the total number of models to four, which will be referred to as MLP (only dense), S_MLP (small, only dense), LIT (literature) and S_LIT (small literature). All models were trained on the same dataset.

**Table 1:** Overview of models, their layers and the number of units/filters for each layer.

LIT	Full-sized units/filters	MLP	Full-sized units
PPG	Conv1: 128 Conv2: 128 Dense: 256	GSR	Dense1: 256 Dense2: 256
GSR	Conv1: 128 Conv2: 128 LSTM1: 256 LSTM2: 256 Dense: 256	PPG	Dense1: 256 Dense2: 256
ET	Conv1: 256 Conv2: 256 Conv3: 256 Conv4: 256 Dense: 1,024	ET	Dense1: 1,024 Dense2: 1,024 Dense3: 1,024
NIRS	Conv1: 512 Conv2: 512 Dense1: 2,048 Dense2: 2,048 Dense3: 2,048	NIRS	Dense1: 2,048 Dense2: 2,048 Dense3: 2,048
HEAD	Dense1: 3,584 Dense2: 4,096 Conv1: 512 Conv2: 512 Conv3: 256 Dense3: 512 Dense4: 256	HEAD	Dense1: 3,584 Dense2: 2,048 Dense3: 1,024 Dense4: 512

*LIT refers to the model that was based on literature, and MLP refers to the model that only contains densely connected layers. Table contains information about the full-sized models, and half-sized models contain exactly half the number of units/filters per layer*.

Two variations of labels were used. The first variation contained samples that were labelled with the indicated difficulty of their respective puzzle and participant, thus containing a total of seven different ‘individual’ labels. e.g., participant 1 indicated a difficulty of 6 for puzzle 3; hence, all samples in puzzle 3 have label 6 for participant 1. Models under this labelling variation were evaluated by their ability to predict what level of workload (LoW) the participant indicated. Participants rated their PMWL on a 7-point scale, seven being the highest PMWL. These ratings were converted to values between 0 and 1 using the formula: (rating 1)/7, such that a rating of 1 corresponded to a label of “0,” a rating of 2 corresponds to a label of “0.1667,” etc. It follows that, in order to be within-level accurate, the average difference between predicted and true labels must be lower than 0.1667. All models used a single output unit with a Sigmoid activation function, resulting in predicted labels between 0 and 1.

The second variation contained samples that were labelled with the average indicated difficulty of all participants over the respective puzzle. This resulted in five different “group” labels, one for each of the puzzles. These ratings were mapped between 0 and 1, where the lowest average rated difficulty corresponded to 0, etc. This labelling variation was used to assess the difference in classification accuracies for individual vs. group labelling schemes. Like the first labelling variation, all models used a single output unit with a Sigmoid activation function, resulting in predicted labels between 0 and 1. An intuitive way of visualising performance was through histograms. By removing the true labels from the predictions, we created a distribution of the error; the ideal result would be a slim Gaussian distribution around zero.

#### Hyperparameter Optimisation

The models were optimised with the help of hyperparameter tuning, or hyperparameter optimisation (HPO). The combination of hyperparameters greatly impacted model performance. In this work, we made use of Optuna, an open-source define-by-run API that allowed us to flexibly and quickly set up a parameter search space (Akiba et al., 2019). We used the default Tree-structured Parzen Estimator to sample values for learning rate, dropout rate and momentum. All losses were calculated as a mean squared error. We opted to use mean squared error because we have ordinal labels ranging from 1–7. Classifying a data sample with label 1 as 7 is a larger error (6 off) than classifying it as a 2 (1 off). Cross-entropy does not take the distance in misclassification into account, whereas MSE does. Furthermore, we provided four different models as categorical suggestions, and these models are discussed in the “Models” section. In total, we ran 20 “trials”; each trial contained a 5-fold cross-validation, in which the total dataset was split into four training and one testing part for every fold. On every fold, a different split was made, and a new model was trained to prevent exposing any trained model to the entire dataset simultaneously. The objective of the HPO was to minimise the average difference between predicted and true labels. To further optimise the learning, we used a learning rate policy that is based on the “1Cycle Policy” as described in Smith (2018). This policy slowly increases and decreases the learning rate in a pyramidal shape as a network cycles through a dataset. This helps prevent getting stuck in local minima. For the creation of visualisations, the best performing models were trained separately using the hyperparameters that were found during HPO. During training, the dataset was split 90–10% train-test randomly, in order to prevent testing the network on previously seen samples. All training was done on a single NVIDIA GeForce GTX 1080-Ti GPU. For further details on the HPO and its implementation, kindly refer to the code that can be found on the following doi: 10.5281/zenodo.4043058 (Dolmans, 2020), or on GitHub^1^.

## Results

In this section, we discuss the retrieved data and the achieved results for the various configurations of models.

### Data

The total number of samples that were selected is 4,082, with an average of 185.5 samples per participant (max: 345, min: 77). The distribution of samples across LoWs can be seen in Table 2. Participants most commonly indicate that the puzzles have a 5/7 difficulty, followed by a 6/7 difficulty. Participants rarely indicate that the puzzles are the easiest (1/7) or hardest (7/7) possible difficulties: 2.1 and 9.3%, respectively. The internal consistency of labels was assessed using Cronbach’s alpha (Tavakol and Dennick, 2011). There were two perspectives from which an alpha could be calculated for the labels. The first was how internally consistent the puzzles are as a predictor of PMWL under the assumption that the puzzles are test items; the resulting alpha was 0.74. The second perspective was how internally consistent the participants are in self-assessing MWL under the assumption that participants are test items; the resulting alpha was 0.97.

**Table 2:** Drift, sample distribution, and puzzle difficulties.

Drift	Difficulties: number of samples	Average puzzle difficulties
Avg: 548 ms	1: 87 (2.131%)	VLow: 2.73
Std: 590 ms	2: 350 (8.574%)	Low: 3.73
	3: 479 (11.73%)	Mid: 4.7
	4: 611 (14.97%)	High: 3.89
	5: 1,275 (31.23%)	VHigh: 5.76
	6: 902 (22.10%)	
	7: 378 (9.260%)	
	Total: 4,082	

*The first column details the recorded drift in terms of the average and standard deviation. The second column summarises the samples and their distribution within each level of difficulty. The third column contains the average difficulty rating for each puzzle on a 7-point scale, as rated by the participants*.

### Model Performance—Individual Labels

As discussed previously, models were evaluated by their ability to predict the LoW the participant indicated. Two sets of 10 trails were done using the Optuna toolbox, once with the MLP and LIT models and once with the S_MLP and S_LIT models. Table 3 contains an overview of the trials that were done on personal labels. The best result that was achieved with the MLP model is an average absolute difference 0.1892 between predicted and true labels. This corresponded to 1.13 LoW when translated back to the 7-point scale that participants rated their PMWL on. Training times per 5-fold cross-validation with 25 epochs per fold were around 40 min. The best result that was achieved with the LIT model is 0.1978, or an average of 1.19 LoW. Training times lay around 70 min. The best result that was achieved with the S_MLP model is 0.1642, or an average of 0.985 LoW. This is also the best result that was achieved within our search space. Training times lay around 25 min. The best result that was achieved with the S_LIT model is 0.1681, or an average of 1.009 LoW. Training times lay around 43 min.

**Table 3:** Overview of hyperparameter optimisation (HPO) trials and their respective scores, ran on individual labels.

Trial	Model	Difference	LoW	Duration (min)
1	MLP	0.5996	3.60	39:09
2	MLP	0.3556	2.13	39:04
3	MLP	0.3685	2.21	39:14
4	MLP	0.2208	1.32	39:18
5	LIT	0.2440	1.46	1:15:49
6	LIT	0.3772	2.26	1:10:32
7	**MLP**	**0.1892**	**1.14**	38:50
8	LIT	0.5672	3.40	1:09:05
9	LIT	0.3798	2.28	1:09:38
10	**LIT**	**0.1978**	**1.19**	1:09:33
11	S_LIT	0.2957	1.77	43:03
12	S_MLP	0.1840	1.104	25:30
13	S_LIT	0.5930	3.558	47:39
14	S_MLP	0.1772	1.063	25:30
15	S_LIT	0.1715	1.029	47:30
16	S_MLP	0.1701	1.021	25:31
17	**S_MLP**	0.1642*	0.985*	25:21
18	**S_LIT**	**0.1681**	**1.009**	47:09
19	S_MLP	0.1808	1.085	25:13
20	S_LIT	0.4534	2.720	47:14

*Trial indicates the trial number of the HPO. Model indicates which neural network was used in the trial. Difference indicates the mean difference between true label and predicted label. LoWs (levels of workload) indicate the number of LoW the mean difference translates to, one LoW being 0.1667. Duration is time needed for a five-fold cross-validation. Bolded numbers are the best performances for each model type. An asterisk indicates best overall performance*.

**Figure 6:**
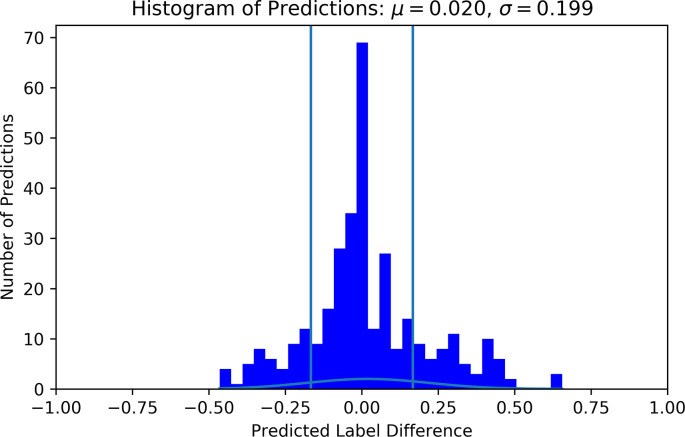
Histogram of predicted vs. true label difference using individual labels. Labels and predictions have been offset such that all true labels are 0 and their respective predictions are deviations away from 0. The vertical lines indicate −1/6 and 1/6 bounds, or one LoW on each side. Predictions outside these lines are more than one workload level inaccurate. For this histogram, the S_MLP was used with HP corresponding to trial 17 in Table 3.

**Figure 7:**
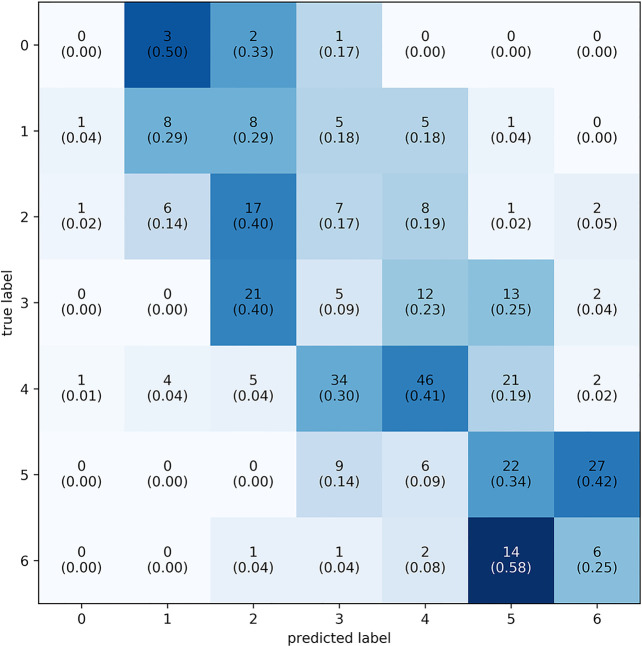
Confusion matrix of the predicted vs. actual classes using individual labels. Labels range from 0–6, totalling seven levels of workload. Every square contains two numbers: the number of times the label was predicted and the relative proportion of predictions in their respective class (in parenthesis). To plot this confusion matrix, predicted labels were placed into the nearest class. The majority of prediction is on or above the diagonal.

The best performing model predicted 63.6% of the samples within one LoW and 72.7% within 1.5 LoW. The distribution of the difference between the predicted and true labels had μ = 0.033 and *σ* = 0.233, see Figure 6. The mean of the distribution was slightly larger than zero, meaning that the model was prone to overestimating the workload of the participant. The confusion matrix shows that the model most frequently correctly classified samples with a label of 0.6667, corresponding to a difficulty rating of 5. See Figure 7 for the confusion matrix. From this confusion matrix, it can be deduced that the accuracy of the classifier is 32%, which is considerably above chance level: a random classifier for seven target labels would correctly classify 14% of the samples. If one considers a difference of one label to also be correct, the accuracy of the classifier is 77%. In this case, a random classifier would have a performance of 3/7 that equals 43%.

### Model Performance—Group Labels

**Figure 8:**
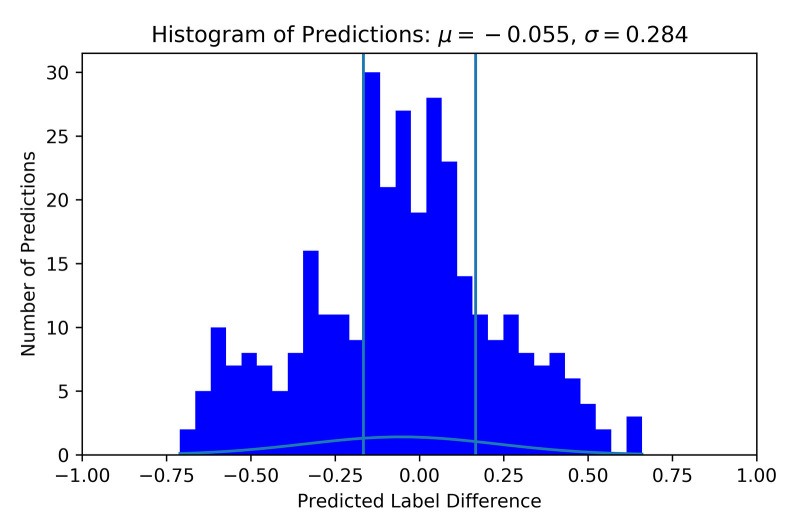
Histogram of predicted vs. true label difference using group labels. Labels and predictions have been offset such that all true labels are 0 and their respective predictions are deviations away from 0. μ indicates the mean, and σ indicates the standard deviation. For this histogram, the S_LIT was used with HP corresponding to trial 16 in Table 5. Vertical lines show the original bounds of 1 LoW for reference.

**Figure 9:**
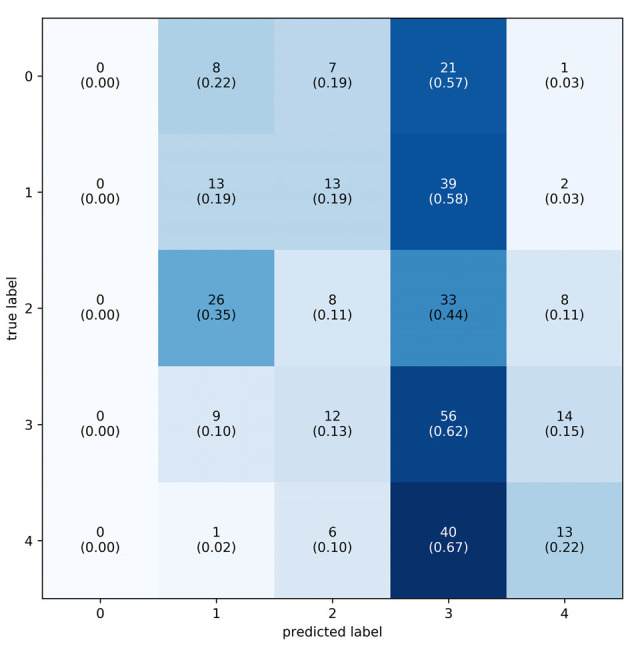
Confusion matrix of the predicted vs. actual classes using group labels. Labels range from 0–4, totalling 5, one for each puzzle difficulty. Every square contains two numbers: the number of times the label was predicted and the relative proportion of predictions in their respective class (in parenthesis). To plot this confusion matrix, predicted labels were placed into bins that represent their class. The majority of predictions are in the second highest workload level.

As discussed, a second labelling variation was assessed. This variation leads to five different labels, and their distribution can be viewed in Table 4. Since these labels depended on the average rating of participants, they were not equidistant. There existed a gap of 0.33 from the lowest difficulty puzzle to the next puzzle. The puzzles thereafter only had 0.05 LoW between them. Similar gaps are present between the third and fourth and fourth and fifth difficulties. Similar to model training with individual labels, a total of 20 trials of HPO were done using the Optuna toolbox. Table 5 contains the results of these trials. The best result was achieved with the S_LIT model, with a mean difference between true label and predicted label of 0.2386. The distribution of prediction vs. label had μ = −0.055 and σ = 0.284, see Figure 8 for clarification. The confusion matrix shows that the model most frequently classifies data into the fourth difficulty, regardless of true label, see Figure 9 for details. In this case, the accuracy of the classifier is 27%, which is just above chance level (accuracy of 20%). If 1 label off is also correct, the performance of the classifier is 72%. Hence, one can conclude that using group labels decreases the performance of the classifier, probably due to the introduction of noise in the labels.

**Table 4:** Labels, occurrences, and prevalence percentages.

Label (puzzle)	Occurrences	Percentage
0 (VLow)	424	10.39%
0.33 (Low)	691	16.93%
0.38 (High)	965	23.64%
0.65 (Medium)	1,138	27.88%
1 (VHigh)	864	21.17%
	Total: 4,082	

*The first column shows the scaled average rated difficulty for the respective puzzle level between parentheses. The second column indicates the number of occurrences for each LoW. The third column details the percentage of occurrences in the respective difficulties*.

**Table 5:** Overview of HPO trials and their respective scores, ran on group labels.

Trial	Model	Difference	Duration (min)
1	**LIT**	**0.2491**	39:09
2	S_MLP	0.2583	39:04
3	**S_MLP**	**0.2484**	39:14
4	S_MLP	0.2642	39:18
5	S_MLP	0.4670	1:15:49
6	S_MLP	0.2540	1:10:32
7	S_MLP	0.3202	38:50
8	S_MLP	0.5389	1:09:05
9	S_MLP	0.2616	1:09:38
10	**MLP**	**0.2616**	1:09:33
11	S_MLP	0.2594	43:03
12	S_LIT	0.2352	25:30
13	S_LIT	0.2546	47:39
14	S_LIT	0.3129	25:30
15	S_LIT	0.2396	47:30
16	**S_LIT**	0.2304*	25:31
17	LIT	0.4366	25:21
18	S_LIT	0.2447	47:09
19	MLP	0.5276	25:13
20	S_MLP	0.2804	47:14

*Trial indicates the trial number of the HPO. Model indicates which neural network was used in the trial. Difference indicates the mean difference between true label and predicted label. Duration is time needed for a five-fold cross-validation. Bolded numbers are the best performances for each model type. An asterisk indicates best overall performance*.

### Unimodal Performance

To investigate the additional value of additional modalities, individual modalities were also evaluated. All models were trained using the hyperparameters that proved most effective for each model type, using individual labels. In other words, HPO was not run for each of the unimodal problems, but relied on earlier optimisations for the respective models. Table 6 details the results of these tests. The best performance was achieved using the PPG modality and the S_MLP model, with an average absolute difference between predicted vs. true label of 0.1969, or 1.18 LoW. The best overall performance for a single modality was achieved with the S_LIT model and the GSR; a difference of 0.1796, or 1.08 LoW. The fNIRS modality performed worst on both models, reaching a difference of 0.2865, or 1.71 LoW, and 0.3188, or 1.91 LoW, for S_MLP and S_LIT, respectively.

**Table 6:** Overview of results for individual modalities.

Model	Labels	Difference	LoW
MLP	Individual	0.1892	1.14
S_MLP	Individual	0.1642	0.985**
LIT	Individual	0.1978	1.19
S_LIT	Individual	0.1681	1.09
MLP	Group	0.2616	–
S_MLP	Group	0.2616	–
LIT	Group	0.2484	–
S_LIT	Group	0.2304	–
	**Modality**		
S_MLP	PPG	0.1969	1.18
	GSR	0.2160	1.30
	NIRS	0.2865	1.71
	ET	0.2159	1.30
S_LIT	PPG	0.2400	1.44
	GSR	0.1796	1.08*
	NIRS	0.3188	1.91
	ET	0.2224	1.33

*The first column contains the name of the model. The second column describes which labels were used. The third column reports the achieved mean difference between predicted vs. true label. The last column shows the mean difference in level of workload. An asterisk indicates the best performance in the unimodal setting; a double asterisk indicates the overall best performance*.

## Discussion

### Performance

Model performance on models trained with individual labels reached sub-level accuracies on a seven-level scale in the classification of PMWL on unseen samples. This showed that the choice of implementation and the IFMMoN were able to learn and generalise from the training data. The histogram shows a normal distribution where the vast majority of points lie around zero, indicating that predicted labels were frequently close to the true labels. The confusion matrix showed that the average prediction of the IFMMoN was slightly higher than the true label, yet a diagonal trend could still be observed. The model performed best in the classification of the fifth LoW, followed by the third and sixth. This followed the trend where more prevalent labels show better performance, with the exception of the third LoW. Furthermore, the fourth LoW, though third-most represented in samples, showed the worst results for unknown reasons. Because of the sample distribution, performance in the relatively underrepresented extremes was hard to assess. This distribution also led to great variability in performance during HPO since the dataset was shuffled differently on each fold. Hence, some folds contained relatively many samples from underrepresented classes in the test set.

The classification of the alternative labelling variation showed no significant diagonal trend; the IFMMoN classified the majority of samples in the fourth LoW, regardless of label. This LoW was the most prevalent label, meaning that the IFMMoN was unable to generalise and minimise loss by classifying into the class that five was the lowest loss. A reason for this could be that the average PMWL did not represent the individual PMWL of participants well enough, rendering the connection between sample and label insignificant. The IFMMoN might learn individual physiology given an individual label but might be unable to generalise in the dataset if all labels are common for vastly different physiologies. In future works, determining the effect of individual differences would be worth pursuing. Model affectivity, as well as performance in general, may vary between subjects, and gaining more insight into these differences can improve the usability of the IFMMoN.

Evaluation of unimodal performance showed that some modalities performed significantly better than others. Though not visually reported in this work, unimodal classification showed a similar trend to the alternative labelling variation: all classifications merge towards one label. This reduces the credibility and value of the classifications based on a single modality. In particular, the fNIRS modality performed poorly. This could mean that the data do not contain valuable signals, or that the implemented processing was inadequate. Another reason for the poor performance of the modality could be the stimulus presentation. The fNIRS modality is often only used in block designs; this optimises the separability of conditions, which is known from the field of functional MRI (fMRI), since the two modalities essentially measure the same signal (Maus et al., 2010). However, stimulus presentation in our research did not follow such a block design. Instead, our work uses a more naturalistic stimulus in that participants worked on several longer tasks. This ‘realness’ of the stimulus allowed us to assess the effectiveness of fNIRS in non-lab situations but likely also negatively impacted the distinguishability between conditions. Because HPO was not done for each of the unimodal problems, but the best parameters for the respective network type were used, performance may not be optimal. In order to determine qualities, such as the informativity of the individual modalities, HPO will have to be done.

To really prove that a multimodal approach outperforms, one needs to validate this on different workload paradigms, such as N-back and visual information overload. Moreover, one needs a sound statistical methodology for proving significance that also includes a false discovery rate correction due to multiple testing. We would not be surprised that for some workload situations, the unimodal approach is on par with a multimodal approach. This however, lies outside of the scope of this research and is a line of further research. While lacking in efficacy, unimodal evaluation allowed us to assess the MG of the implementation. Altering the configuration of the IFMMoN was quick and simple due to the modular design. This indicated that the modularity criterium was adhered to, and that it was practical during research. Furthermore, since we were able to achieve better performance using all modalities, the generalisability criterium was also satisfied. The IFMMoN appeared to generalise better given data from multiple physiological sources. Hence, we consider the MG criteria to be practical and valuable.

### Limitations

Our limitation is that we are aware of some in several categories. First, there are hardware limitations, which become most apparent when inspecting device synchrony. The average recorded drift across all participants was 548 ms (SD = 590 ms, max = 2, 827 ms, min = 58 ms). In total, four participants had a drift of larger than 1 s. For two of these, the reason is known: crashing software and device shutdown before recording end. The reason for the drift in the remaining two is unknown. An average recorded drift of 548 ms is quite large for some modalities, such as ET, and not so much for fNIRS. If this system is to be used with a modality that is even more sensitive to drift, such as electroencephalography (EEG), significant improvements need to be made. One way of doing this is by performing data collection on a more powerful computer, or by distributing the data collection over multiple computers. Dedicating more CPU to each device and stream will likely yield better results. Device-specific hardware limitations may also play a role in the drift of data streams. Finally, the recording software may also be looked to when investigating the drift further.

Second is the limited number of participants. A common way of improving performance is by gathering more data. In total, 4,082 samples were collected from 22 participants. To put this dataset in perspective, ImageNet, a large image database that is commonly used, has over 14 million images (Deng et al., 2009). Of course, gathering physiological data is significantly more time-consuming, especially when using multiple devices. Nonetheless, an almost-guaranteed way of improving the performance is to gather data from more participants.

Third is the choice of modalities. Currently, only gaze data (X and Y coordinates of both eyes) are used in this work. Duchowski et al. (2018) demonstrate the efficacy of pupillary activity with regard to the assessment of cognitive load. The inclusion of pupillary data into this work may have led to different results. The same can be said for our measurements of the brain. Currently, fNIRS is used to measure the relative changes in (de)oxyhaemoglobin. However, EEG can also be used to predict cognitive load, as demonstrated by Friedman et al. (2019). This train of thought can be extended to other measures, to the extent where this same research can be performed with a different set of modalities to achieve vastly different results.

A fourth limitation is the model architecture and optimisation. Currently, two variations of IFMMoN were used, each with a small and large version. Smaller versions showed better performance while also being more efficient, possibly due to the small size of the dataset. No further exploration into where model performance stops improving with the reduction of model size and/or complexity was done. Furthermore, HPO was done only on momentum, learning rate and dropout rate. This can be improved by also varying the number of hidden layers and neurons, as demonstrated by Akiba et al. (2019). On the other hand, the performance estimates given in the results could be a little optimistic due to the fact that HPO applied was on the total dataset instead of using nested cross-validation (i.e., applying HPO on each train fold in the cross-validation approach). But, since HPO was used to optimise only some learning parameters, the presented performances are a good reflection of the actual performances.

Data selection around markers can be changed and customised for each of the modalities. For example, selecting fNIRS data around marker can be done differently when compared to ET data, given that the haemodynamic response is very ‘slow’ compared to eye movements. For this reason, data after the markers could also contain valuable information for some modalities. Worth noting, however, is that changes to the dataset or modalities would require the network to be retrained and HPO would need to be redone. If a modality is added, then the Head network and the sub-network corresponding to the new modality need to be (re-)trained. If a modality is removed, then only the Head network needs to be re-trained. This is a time- and resources-consuming process. Testing on additional subjects that contain the same modalities does not require retraining. The latter is something that was not tested in this research and is thus considered one of its shortcomings. Lastly, network outputs could be encoded in a 7-dimensional vector where each output gives the probability of this the respective label, rather than outputting a single number between 0 and 1.

Finally, some complications arose during collection and upon inspection of the retrieved data. Data of Participants 3, 8, 13 and 16 were partially excluded due to software crashes and poor device connectivity. Participants 6, 9, 15 and 21 sporadically show minor artefacts likely related to movement or dark hair. However, this data was included in the dataset with the intention to expose the system to a certain, perhaps more realistic, degree of noise.

### Labelling

A more psychometric point of discussion lies in our labelling scheme. Participants will give different ratings for the same PMWL. Ratings are entirely subjective and volatile because one can only assess one’s PMWL relative to oneself. Furthermore, one person might feel confident and calm while experiencing high workload, whereas another might feel stressed while experiencing low workload. Hence, one should always expect to see a high degree of error and variance when assessing PMWL, or any human emotion for that matter. Since the objective of our system is to eventually classify PMWL in naturalistic environments in real-time, we chose to work with such naturalistic stimuli from the start. Comparing classification results between our labelling variations that accuracy is highly sensitive to which labelling scheme is used. Based on these observations, our recommendation is to use individual labels to train the IFMMoN.

## Conclusion

The goal of this research was to use PMWL using a multimodal DNN. While participants were solving verbal logic puzzles, GSR, PPGF, fNIRS and ET data were collected simultaneously using LSL. We proposed a novel IFMMoN; the best model was able to classify PMWL with a 0.985 LoW accuracy on a 7-level scale. This result allows us to conclude that the IFMMoN can use the provided four modalities to classify PMWL. The MG criteria were guiding in all stages of the research: data collection, data selection and model design. The modularity criterium was satisfied through streaming of data from various separate applications into one collection software, as well as the choice of intermediate fusion using MNets that feed into one Head model. Generalisability was satisfied through improved model performance when adding multiple modalities. We showed that smaller models achieved better results in our classification task, while experiencing a speedup factor roughly equivalent to the size-difference factor. A critical discussion highlights the strong and weak points of this work, and we highlight clear avenues for improvement. Future works will work towards the classification of PMWL in real-time so that applications can be adapted to their users.

## Future Works

Currently, two variations of labels were trained on: one with individual difficulty ratings and one with averaged difficulty ratings over all participants. However, the output of our models is always a number between 0 and 1. Different objectives for classification can be interesting to pursue. Given a known option space, a vector containing probabilities of a participant’s next move can be outputted. This could be interesting because it would allow the prediction and even interception of mistakes. A different route would be to train on data that originate in an alternative task. This would yield insight into the generalisation capabilities of the pipeline and networks and would thus likely benefit the overall robustness of the system.

The long-term outlook of this line of research is to create a system that can classify user PMWL in real-time and eventually can do so for multiple users simultaneously. Users can then be steered to improve the overall efficacy in their task, whatever it may be. This can, for example, be done by adapting the environment’s intensity to trigger a state of flow. However, the participant can also be adapted to the environment by modulating the participant. Such modulation can be done with the help of visual, audial or even olfactory stimulation (Hughes, 2004; Weinbach et al., 2015). This research takes several relevant steps in the direction of such a system since it shows that PMWL can be classified accurately using multiple modalities. Additional modalities and users can easily be added due to the employed design principles. Furthermore, the size of the network allows for real-time implementation.

Moreover, such a system might even be able to detect which person is currently using it. This can improve the system’s adaptability, as well as clusters usage patterns, similar to what is done in unsupervised problems. The ability to cluster users together may prove especially valuable in collaborative and team contexts. McDonald and Solovey (2017) demonstrated the potential of using fNIRS to distinguish between 30 different users with 63% accuracy, providing a clear route for implementation.

### Data Augmentation

A proven method of increasing model accuracy is simply to supply more data, so that the model is better able to generalise. However, the task of collecting, formatting and labelling data is time-consuming and expensive. Data augmentation allows for the generation of new and unseen data, offering a solution to the data shortage problem. There are several different options for data augmentation. It can be done in the input space, the feature space or in the learned feature space, to name a few. Augmentations in the input space involve performing several transformations on the original data. In image classification, this often takes the form of rotation or scaling, or by adding noise to the image (Sajjad et al., 2019; Sun et al., 2019). For data augmentation in input and feature space, domain expertise is often required to ensure that newly generated data respects the domain from which it is synthesised. Examples are works by Steven Eyobu and Han (2018) and Schlüter and Grill (2015). Generative models were also proven to be capable of performing such tasks while also overcoming missing data and even modalities (Ngiam et al., 2011; Srivastava and Salakhutdinov, 2012). In the above examples, new feature extraction and augmentation blocks must be designed for each data type and problem specifically. This requires both tailoring, as well as domain expertise, and therefore does not generalise well across domains and problem statements.

Vries and Taylor propose to perform data augmentation in the learned feature space (DeVries and Taylor, 2017). Their approach relies on first learning a representation of the data and then performing data augmentations on those representations. They hypothesise that simple augmentations on encoded data, rather than input data, result in more plausible synthetic data. They propose using a sequence autoencoder on the bases of the proven generalisability of the seq2seq models that were independently devised by Cho et al. (2014) and Sutskever et al. (2014). The approach that DeVries and Taylor (2017) propose has several benefits over the other discussed methods of data augmentation. Similar to a previous work (Sutskever et al., 2014), the feature augmentation is done in reduced dimensionality, making the implementation lightweight. However, instead of designing constraints that are specific to the domain task and input data, more generalised parameters that dictate augmentation can be formulated. These parameters are then also eligible for HPO. Hence, this approach fits best within the MG criteria and would be worthy of pursuing in future works.

## Data Availability Statement

The datasets presented in this study can be found in online repositories. The names of the repository/repositories and accession number(s) can be found below: 4TU.ResearchData; doi: https://doi.org/10.4121/12932801.

## Ethics Statement

The studies involving human participants were reviewed and approved by University of Twente Behavioural, Management and Social Sciences Ethics Committee. The patients/participants provided their written informed consent to participate in this study.

## Author Contributions

TD: ideation, execution of research, data collection and processing, manuscript writing, dataset and code publication. MP: feedback, supervision and funding. J-WK: feedback and supervision. BV: feedback and supervision. All authors contributed to the article and approved the submitted version.

## Conflict of Interest

The authors declare that the research was conducted in the absence of any commercial or financial relationships that could be construed as a potential conflict of interest.

## Footnotes

https://github.com/Tech4People-BMSLab/mwl-detection.
